# Patients with acute exacerbation of chronic obstructive pulmonary disease feel safe when treated at home: a qualitative study

**DOI:** 10.1186/1471-2466-12-45

**Published:** 2012-08-24

**Authors:** Ying Wang, Torbjørn Haugen, Sissel Steihaug, Anne Werner

**Affiliations:** 1 HØKH, Research Centre, Akershus University Hospital, P.O. Box 95, N-1478, Lørenskog, Norway; 2 Faculty Division Akershus University Hospital, University of Oslo, Oslo, Norway; 3 Clinic for Allergy and Airway Diseases, Oslo, Norway; 4 SINTEF Technology and Society, Health Research, P.O. Box 124, Blindern, N-0314, Norway

**Keywords:** Hospital at home treatment, Acute exacerbation of COPD, Qualitative interviews, Patient perspective

## Abstract

**Background:**

The design of new interventions to improve health care for patients with chronic obstructive pulmonary disease (COPD) requires knowledge about what patients with an acute exacerbation experience as important and useful. The objective of the study was to explore patients’ experiences of an early discharge hospital at home (HaH) treatment programme for exacerbations in COPD.

**Methods:**

Six exacerbated COPD patients that were randomised to receiving HaH care and three patients randomised to receiving traditional hospital care were interviewed in semi-structured in-depth interviews. Four spouses were present during the respective patients’ interviews. The interviews were audio-taped, transcribed and analysed by a four-step method for systematic text condensing.

**Results:**

Despite limited assistance from the health care service, the patients and their spouses experienced the HaH treatment as safe. They expressed that information that was adapted to specific situations in their daily lives and given in a familiar environment had positive impact on their self-management of COPD.

**Conclusion:**

The results contribute to increased knowledge and awareness about what the patients experienced as important aspects of a HaH treatment programme. How adapted input from health services can make patients with exacerbation of COPD feel safe and better able to manage their disease, is important knowledge for developing new and effective health services for patients with chronic disease.

## Background

Chronic obstructive pulmonary disease (COPD) has become a major cause of morbidity and mortality during the last two decades and is expected to become the third leading cause of death worldwide in 2030 [1]. Acute exacerbation of COPD resulting in hospitalisation is a serious event for patients, and frequent hospitalisations have been related to reduced survival [2] and impaired quality of life [3]. Demographic changes with an increasing number of older people with chronic diseases with the demand for acute care hospital beds can be expected to increase. One way of organising help for some of these patients is to provide hospital at home (HaH) treatment [4].

Different HaH programmes for treatment of acute exacerbation of COPD have been carried out and studied in both randomised controlled studies [5-13], non-randomised studies [14-19] and studies with retrospective analysis [20,21] in countries like the United Kingdom [6,7,12-17], Spain [8,9,19], Australia [10] and Italy [5]. These studies have shown that it is feasible for selected patients with acute exacerbation of COPD to be treated at home, and that the participating patients were satisfied [12,13]. Some studies have found significant cost savings with HaH [9,10,22], but other studies have not been able to confirm this [5,23].

A systematic review from the Norwegian Knowledge Centre for the Health Services concluded that patients with acute exacerbations of COPD, who were randomised to HaH treatment, had a lower readmission rate than patients who were randomised to conventional inpatient hospital treatment [22]. Treatment at home showed a statistically non-significant trend towards lower mortality. Finally, the review concluded that it is uncertain whether patients and next of kin are satisfied with receiving HaH treatment [22].

Some qualitative studies have been carried out on patients’ experiences of HaH programmes. Clark et al. concluded that not all patients found the home nursing component of the services helpful, and that the patients felt that they were not being actively involved in the early discharge process [23]. Schofield et al. on the other hand found that the majority of the patients and their family preferred home care service rather than hospital care [24]. A qualitative study on experiences of patients with COPD who had participated in an extensive self-management treatment intervention including self-management education course and a fitness programme in an outpatient clinic, found that the patients felt safe [25]. However, a detailed description of the patients’ experiences of the home treatment programme is lacking [24,25].

The objective of the present study was to explore COPD patients’ experiences of a limited early discharge HaH treatment programme. We concentrated on aspects of special importance to the patients during and after the acute treatment period. Knowledge about what patients experience as helpful health care services and support is needed in development of new ways of organising and carrying out patient treatment.

## Methods

### Subjects

Patients were recruited from consecutive participants in a randomised controlled trial (RCT), investigating long-term effects of a HaH programme. The main inclusion criteria of the RCT were that the patients were considered to have acute exacerbation of COPD according to the definition adopted by the Global Initiative for Chronic Obstructive Lung Disease (GOLD) [26,27] and needed hospital admission. The main exclusion criteria were life threatening respiratory failure, confusion, impaired consciousness and changes in chest x-ray or comorbidities in need of further inpatient investigation or treatment. Within 36 hours the patients were allocated to HaH or continued hospital treatment. This RCT experienced a slow inclusion rate and was terminated after a year because of this. At this point 12 patients were included.

The 12 patients included in the RCT were considered eligible for inclusion in this qualitative study. After excluding three patients because their medical condition had worsened, all of whom had received in-hospital treatment, nine patients were included (Table 1). Six patients received HaH treatment (HaH patients) and three received traditional hospital treatment (inpatients). The inpatients were interviewed in order to see the HaH patients’ experiences in light of traditional hospital care. Written informed consent was obtained from the participants, and the study was approved by the Regional Committee for Medical Research Ethics (REK), South-East Norway.

**Table 1:** Characteristics

***Hospital at home treatment programme***							***Hospital care***		
**On admission**									
**Sex**	**Female**	**Female**	**Female**	**Male**	**Male**	**Male**	**Female**	**Female**	**Male**
Age (yrs)	51	71	71	70	71	79	54	77	77
Marital status	Married	Widow	Widow	Married	Married	Married	Divorced	Widow	Married
Comorbidities	Anxiety Depression	Heart Disease Hypertension	Heart Disease Hypertension	Hypertension	Diabetes mellitus Hypertension	Diabetes mellitus Hypertension	Anxiety	Diabetes mellitus Inflammatory bowel disease	Heart disease
GOLD stage	II	I	III	III	III	II	III	III	III
Blood pressure (mmHg)	150/80	180/80	140/75	155/90	150/90	140/70	140/75	150/80	97/53
Pulse rate min^-1^	108	56	87	75	88	61	105	110	60
PaO_2_ (kPa)	8.27	8.45	10.9	7.57	11.4	8.93	9.18	8.74	8.13
Respiratory rate min^-1^	24	22	20	35	24	18	17	20	25
Number of drugs	5	8	8	5	8	10	5	8	8
**At 6 weeks control**									
FEV_1_ (l/s) (% of predicted)	1.68 (59%)	1.89 (97%)	0.5 (31%)	1.4 (37%)	1.3 (40%)	2.1 (75%)	0.9 (39%)	0.8 (41%)	1.3 (42%)
MRC dyspnoea scale	5	3	3	2	2	2	3	4	2
BMI (kg/m^2^)	41	33	24	38	33	30	30	26	24
6-minute walk test (m)	*	508	272	500	125	510	280	350	500

IBD: Inflammatory bowel disease; GOLD: Global Initiative for Chronic Obstructive Lunge Disease; PaO_2_: arterial oxygen tension; FEV_1_: forced expiratory volume in one second; MRC: Medical Research Council dyspnoea scale, range (1–5); BMI: Body mass index; *not carried out due to a knee injury.

### The hospital at home treatment programme

A specialised hospital nurse visited the HaH patients up to one hour daily over a period of three days after hospital discharge. The nurse evaluated the patients’ clinical status, essential clinical parameters, obtained blood samples for later analysis when needed, and assessed whether or not the patients could still be treated at home. The nurse could consult a pulmonologist in case of worsening of the patients’ symptoms. After the consultation, decisions were made on frequency of follow-up, therapy changes or readmission to the hospital. Furthermore, the nurse invited the patient and his/her spouse to a dialogue, e.g. she asked how the patient felt and encouraged them to reflect on possible causes of the acute exacerbation and how to prevent it. During the three-day period the patients were allowed to call the hospital at any time if they were concerned about their condition, especially regarding indications for readmission. Inpatients were treated according to ordinary hospital routines.

Both patient groups were during the subsequent year offered three outpatient follow-up consultations with a pulmonologist in the hospital. The first visit was approximately six weeks after discharge.

### Data collection and analysis

The empirical data were obtained from semi-structured, audio-taped in-depth interviews based on Kvale’s principles [28]. The researcher had prepared interview guides for the two informant groups (HaH patients and inpatients) before the interview, but the guides were used flexibly. This implied that all the questions in the guide were asked, but different topics could be explored more deeply in different interviews, and that new topics could be brought up by the informants during the interviews. The interviews included questions about the patients’ experiences and their benefit of the treatment programme. The interviews were carried out in the patients’ home between seven and ten weeks after hospital discharge, except for one interview that was conducted in the outpatient clinic. The interviews lasted between 60 and 90 minutes with the HaH patients, and 30 minutes with the inpatients. Four spouses (three of the HaH patients and one of the inpatients) were present and participated in varying degree. The first author, a medical doctor, carried out and transcribed the interviews. She was trained in asking open-ended rather than closed questions and in asking the participants about not only positive, but also negative experiences with the HaH programme [28]. The analysis was carried out in collaboration with the supervisors.

Interview transcripts constitute our data material. It was analysed according to a systematic text condensing method in the following four steps alternating between the various steps throughout the entire process as described by Malterud [29]: 1) Reading through the whole material to obtain an overall impression; 2) Identifying themes representing different aspects of the patients’ experiences and coding these under different thematic headings (i.e. coding groups). The coding groups “feeling safe,” “individually adapted information,” and “managing strategies” were developed; 3) Abstracting and condensing the content within each coding group; and 4) Summarising the content within each thematic heading. Suitable citations were chosen to illustrate the findings.

The analysis focused on the patients’ experiences of the HaH programme, and whether the patients had obtained something from the programme that helped them to manage their chronic illness in everyday life. This was seen in light of the inpatients’ experiences of traditional hospital care.

## Results

### Feeling safe

The patients spontaneously described the HaH programme as safe, mainly according to the daily visits of the nurse. The patients emphasised different aspects of the programme that contributed to their experiences of feeling safe, e.g. the treatment predictability due to the cooperation with the nurse when deciding the time of the visits. Daily examinations of clinical parameters were experienced as “very reassuring.” One summed it up as the following:

“It was safe, because I knew she was coming! If I did not feel 100% well, I knew that she was coming tomorrow to check me.” (HaH patient 5)"

The patients also pointed out the importance of the possibility of telephone consultation with the pulmonary department and instant readmission when needed.

Several patients drew attention to how reassuring it was to have time for asking questions and talking with the nurse during the visits. One HaH patient said:

“The more you know, the safer you feel. You are not so frightened when you know what is what and get a proper explanation of this [disease].” (HaH patient 1)"

Four patients appreciated that the nurse would ask, “How are you?” They remarked that she was interested in listening to how they felt. Some also mentioned the comprehensible way the nurse had explained things and her high competence that made them feel like they were “in safe hands.” The patients used words such as “very pleasant,” “cosy” and “a caring person” to describe the visiting nurse.

Two patients, both readmitted, said that they retrospectively felt that the discharge from the hospital within 36 hours after admission might be too early. However, both patients would choose home treatment again. A third patient wished for a longer home treatment period and perhaps also more than one visit a day.

Four patients emphasised the significance of being followed up with three controls at the hospital during the subsequent year. This contributed to their experience of the programme as reassuring.

The three inpatients said they were very satisfied with the hospital treatment. They were surprised by being asked whether they felt safe during their hospital stay. They expressed that they automatically felt safe after admitted to the hospital. Two of them said that they immediately felt safe when hospitalised, although their breath is still heavy. The statement of one of the patient’s wife summed up the three inpatients’ experiences as follows:

“I think one feels safe when doctors and nurses are present: I believe it’s quite natural!” (Inpatient 3, spouse)"

### Individually adapted information

The HaH patients emphasised information as important. They valued “getting an explanation” and an understanding of the disease and its consequences. This made them feel safe and calm, as well as less anxious. Four patients and two spouses remarked that the information was detailed and related to concrete situations in their everyday life. One patient stated that being treated at home made it easier to concentrate on her challenges in daily life:

“When you are at home you know what’s what, and may think of various things that are relevant just then. It was just me and her and nothing to disturb us.” (HaH patient 1)"

One patient’s wife appreciated that the nurse had answered several questions concerning her worries for her ill husband, e.g. questions related to breathing, diet and lifestyle. One patient said the nurse had explained many things in a comprehensible way, things she had struggled with without being aware of it:

“She said that I do not need to get everything done in one day even if I am in a good period. I learned to distribute the energy evenly and listen to my body signals. She made suggestions and put me on the track to many good ideas.” (HaH patient 1)"

The nurse had given advice related to how to manage the limitations caused by COPD, be aware of symptoms, e.g. cough, mucus and breathlessness and how to manage them. One patient had been advised to take it easy at the start of physical exertion so that his “breath” would last longer. She also gave advice on dealing with the unpleasant influence of cold air in the winter. Four patients mentioned that the nurse had discussed medication and how to use various medical equipments.

Two patients experienced the nurse’s information as less helpful. One said she already had received similar information at a previous rehabilitation programme. Another patient emphasised that the written information he had received from the nurse was not fitting for his specific case:

“There is something about smoking on every page [of the brochure], but I have never smoked!” (HaH patient 3)"

According to the three inpatients, the information they got during the hospitalisation was related to treatment and results from various medical examinations. The inpatients noticed that the hospital staff was busy, and they did not expect them to have “time for sitting down talking for half an hour,” as one said. They were surprised when being asked whether there was some information they missed. The following quotation summed up their reactions:

“Is it possible to get any more information?” (Inpatient 2)"

### Managing strategies

Three of the HaH patients described different aspects of changes in their everyday life related to the received information, explanations and advice about management of the disease from the visiting nurse. One patient shared how he applied the advices: he had learned to relieve the breathlessness during an acute attack by hanging over the armchair in his living room. Earlier he used to swallow the mucus, but he had begun to cough it up even at night now. In order to breathe easier during the night, he had elevated the head end of his bed.

Two patients said they had improved their routines after receiving the HaH. One used to skip prophylactic medication because she did not immediately experience any effect. After she had been given an explanation of how the medication works, she had begun to take it regularly. Another patient used to take a cough mixture before she went to bed to avoid coughing at night. She stopped taking this drug after having been informed about the negative effects of it. She also felt herself in a better physical and psychological mood after she began to distribute her energy more evenly than before.

The three inpatients did not mention similar experiences from having received information and advice during the hospitalisation. They rather emphasised that they were satisfied with the stay due to the quality of the service from “the nice staff.” One said:

“[The staff] was constantly dropping in to ask if I was thirsty or something…” (Inpatient 2)"

One inpatient’s spouse remarked that she got the impression that the hospital was mostly concentrated on getting patients out.

## Discussion

One main result of this study is that the HaH patients’ feeling of safety was their main experience from the treatment programme and that this was crucial for their positive experience of the programme. This was surprising considering the limited time of professional treatment. The HaH patients felt they were being taken care of due to the daily visits by the nurse, which seems to contrast the results in another qualitative study [23]. Our analysis clearly indicated that the patients’ involvement in deciding the schedule contributed to their feeling of predictability of the treatment programme. That the possibility to call the hospital at any time was highly valued by the patients and probably functioned as a security net, is supported by the results in a qualitative study by Monninkhof et al. [25].

To our knowledge, the importance of feeling safe has previously not been demonstrated in studies on HaH programmes with similar design with relatively limited help as is the case in our study. However, participants in a qualitative study on a considerably more comprehensive programme lasting up to two years expressed that they felt safe [25]. Moreover, two RCTs and one mixed-method study found that the patients were satisfied with and preferred HaH, but it is not in detail described why and what made the participants in their study prefer HaH [12,13,24].

An important finding in our study was that the HaH patients appreciated the individually adapted information they got, including those patients, who previously had undergone a four weeks in-hospital rehabilitation.

The treatment programme took place and the information was given in the patients’ home and this made it easier for them to participate in the treatment process, in contrast to passively waiting to be helped, as the inpatients experienced. The home environment provided a unique framework for the treatment and the information. The patients were reminded about what they used to struggle with in their everyday life and the nurse could adjust her answers, information and advice to the concrete situations and objects in the patients’ home and teach them how to manage their disease. The information was therefore related to the patients’ needs and easy to remember and practice afterwards.

Another important aspect of the HaH was that the nurse had enough time to talk with one patient at a time without distraction, which routines in the hospital rarely allow. This made it possible for the nurse to practise her role as an adviser, treatment manager and teacher. It is likely that self-management education is beneficial for patients with COPD in a way that the patients apply the knowledge from the education to guide self-management over time [30]. Our findings show that the patients appreciated the calm way the nurse worked, without signalling being in a hurry, as also previously reported to be valued in another study [24]. In contrast, the inpatients’ stories reflect how modern care services can be negatively interpreted. The health professionals were perceived to be nice and trying to do a good job, but their work was understood to be distracted by strict routines and technological procedures. The inpatients experienced that the staff was always in a hurry and seemed to have little time for communicating with the patients, but nevertheless appeared to do their best.

During the last five decades chronic diseases are major causes of disabilities, and the aim of the treatment of a chronic disease is to improve health related quality of life and maintain independency for the patients [30]. In the case of COPD, self-management is an important part of living with this disease [31]. To teach patients skills needed to carry out medical regimens and help them manage their illness in everyday life are essential parts of the treatment. Our findings indicated that the individualised information and instructions from the nurse, given in the patients’ homes and in connection to their everyday life situations contributed to the patients’ experiences of having control and feeling safe.

We chose the qualitative in-depth interview as a method to develop knowledge of the patients’ experiences of the HaH. Six HaH patients, selected through the inclusion criteria to the RCT, may seem to be a small number, but is not unusual few informants in a qualitative study [29,32,33]. Although we did not manage to include more than six patients, we had god diversity in our materials as regards age, gender, marital status and GOLD-stage among our subjects (Table 1). In this selected group of patients with COPD, we discovered broadness in the patients’ experiences, which we found comparable to studies with higher numbers of patients [24,25].

Additionally, we consider the research method well suited in the sense that we got rich information and new knowledge despite that some patients had difficulties in remembering detailed information. Interviews earlier than seven to ten weeks after the treatment may have reduced the recall problems in some degree. We believe the involvement of the spouses in the interviews was a valuable informational supplement and interviews with the inpatients shed light on the specific aspects of the HaH.

## Conclusion

The patients in this study experienced the HaH programme as safe despite the limited help they received. They expressed that information that was adapted to specific situations in their daily lives and given in a familiar environment had positive impact on their self-management.

How adapted input from health services can make patients with exacerbation of COPD better able to manage their disease, is important knowledge for developing new and effective health services for patients with chronic disease.

## Competing interests

The authors declare that they have no competing interests.

## Authors’ contributions

YW: contributed to design the study, and is main responsible for data collection, analysis and interpretation of data, and drafting of the manuscript. TH: contributed to design the study, and critically revised the article. SS: contributed to the data analysis and critically revised the article. AW: contributed to the study design, data collection, analysis and interpretation of data, drafting, and critically revising the article. All authors read and approved the final manuscript.

## Pre-publication history

The pre-publication history for this paper can be accessed here:

http://www.biomedcentral.com/1471-2466/12/45/prepub
